# Beaver Tail Liver Masquerading As Acute Pancreatitis

**DOI:** 10.7759/cureus.63015

**Published:** 2024-06-24

**Authors:** Harshitha Reddy, Shilpa A Gaidhane, Sunil Kumar, Manjeet Kothari, Suprit Malali

**Affiliations:** 1 Department of Medicine, Jawaharlal Nehru Medical College, Datta Meghe Institute of Higher Education and Research, Wardha, IND

**Keywords:** computed tomography abdomen, alcoholic hepatitis, case report, acute pancreatitis, beaver tail liver

## Abstract

An unusual anatomical variation known as "beaver tail liver" occurs when the liver's left lobe spreads laterally until it touches the spleen. It is also known as a sliver liver, saber-shaped liver, or flax-like liver. We are talking about a 34-year-old man, a chronic alcoholic in this case, who had complaints of upper abdominal pain, persistent vomiting, and abdominal palpation elicited tenderness in the upper abdomen. Also, he had hepatosplenomegaly. On further investigation, he was diagnosed with alcoholic hepatitis, and on computed tomography, there was enlargement of the left lobe of the liver, which was beaver tail liver. This case report aimed to present a detailed account of a patient presenting with upper abdominal pain and clinical suspicion of acute pancreatitis. On imaging, there was a beaver tail liver. This unusual morphology can be an incidental finding during imaging studies or surgical procedures, often posing diagnostic challenges and considerations for clinicians.

## Introduction

The term "beaver tail of liver" refers to a variant of the standard hepatic form in which the liver's left lobe expands to touch and often encircles the anterior portion of the spleen. It is also known as a sliver liver, saber-shaped liver, or flax-like liver. This finding is rare and is more common in females [1]. Typically, beaver tail liver is discovered by chance during abdominal imaging. The parenchyma bears the same risks of hepatic pathology as the rest of the liver, with the probable exception of damage resulting from trauma to the lower left chest or left upper quadrant. It may be challenging to distinguish between the liver and spleen when their computed tomography (CT) densities or ultrasound echogenicities are the same. Even if their density or echogenicity appear different, they can be confused for a splenic mass or a perisplenic/subcapsular hematoma [2].

Alcoholic hepatitis is an acute inflammatory condition of the liver induced by excessive alcohol consumption, characterized by hepatocellular injury, inflammation, and fibrosis. This condition can present with a spectrum of symptoms ranging from asymptomatic liver enzyme elevations to severe liver dysfunction, manifesting as jaundice, coagulopathy, and hepatic encephalopathy [3]. The pathogenesis of alcoholic hepatitis involves complex interactions between ethanol metabolism, oxidative stress, immune responses, and genetic predispositions. Jaundice, malaise, painful hepatomegaly, and mild indications of an inflammatory response are typical symptoms of alcoholic hepatitis. Aspartate aminotransferase (AST) >50 U/L and never more than 400 U/L, serum total bilirubin above 3 mg/dL, and a De Ritis above 1.5 are found in the investigations. This illness is characterized by fever, tachycardia, tachypnea, hepatomegaly, and chronic alcohol usage up to four weeks before the beginning of symptoms [4,5].

Beaver tail liver, though often asymptomatic, can pose challenges in diagnosis and surgical procedures. This anatomical anomaly can present with symptoms and imaging findings that mimic those of acute pancreatitis, posing a significant diagnostic dilemma. The rarity of this variant should increase awareness among clinicians to ensure appropriate management and avoid complications during hepatic or abdominal surgeries [5,6]. Based on clinical signs, the patient in this case report was initially believed to have acute pancreatitis, but further investigation revealed that the actual cause was alcoholic hepatitis with a beaver tail of the liver, an unusual hepatic morphology causing pain in the upper abdomen mainly in the epigastrium and radiating to the back. This instance emphasizes how crucial it is to identify liver structural differences that can mimic other abdominal illnesses.

## Case presentation

A 34-year-old male came to the outpatient clinic reporting sudden onset pain in the upper abdomen, most intense in the epigastric region, rated 9/10 in intensity, and radiating to the back. He also complained of persistent vomiting, occurring five to six episodes per day for three days, which was aggravated by food intake. He had been a chronic alcoholic for 10 years and has been binge drinking about 500 mL/day of country liquor for the past seven days. The patient had no notable medical history or symptoms such as fever, black-colored stools, hematemesis, headache, visual disturbances, seizures, trauma, chest pain, breathlessness, fever, or orthopnea. The patient had no comorbidities like hypertension and diabetes.

During the physical examination, the patient’s temperature was 97.9°F, blood pressure 130/80 mm Hg, pulse rate 120 beats/minute, respiratory rate 24 breaths/minute, and SpO_2_ 98% on ambient room air. The patient had icterus, and there were no signs of pallor, clubbing, cyanosis, lymphadenopathy, and pedal edema.

Gastrointestinal system examination revealed tenderness in the epigastrium, left and right hypochondrium, and hepatosplenomegaly. There was guarding in the upper abdomen. Other systems were normal on examination. Laboratory investigations are shown in Table 1.

**Table 1:** Laboratory investigations on the day of admission ALT: alanine transaminase; AST: aspartate transaminase

Lab investigations	Observed value	Normal lab values
Hemoglobin	8.3	13.2-14.7 g/dL
White blood cell count	11,200	4,000-11,000/mm^3^
Platelets	1,65,000	1,50,000-4,50,000/mm^3^
RBC count	2.34	4.2-5.5 million/mm^3^
Mean corpuscular volume	106.7	78-101 fL
Blood urea nitrogen	5	4-10 mg/dL
Creatinine	0.6	0.6-1.0 mg/dL
Potassium	3.3	3.5-5.1 mmol/L
Sodium	143	135-145 mmol/L
ALT	46	<50 U/L
AST	191	17-59 U/L
AST:ALT ratio	4.15	>1.5
Alkaline phosphatase	179	38-126 unit/L
Total protein	7.8	6.2- 8.3 g/dL
Albumin	2.9	3.3-5.2 g/dL
Total bilirubin	7	0.2-1.2 mg/dL
Conjugated bilirubin	4.9	0-0.3 mg/dL
Unconjugated bilirubin	2.1	0-1.1 mg/dL
Lipase	244	23-300 u/L
Amylase	60	30-110 u/L
International normalized ratio	1.7	0.8-1.2
Prothrombin time	12.9	11.9 control
Activated partial thromboplastin clotting time	34.6	29.5 control

**Figure 1:**
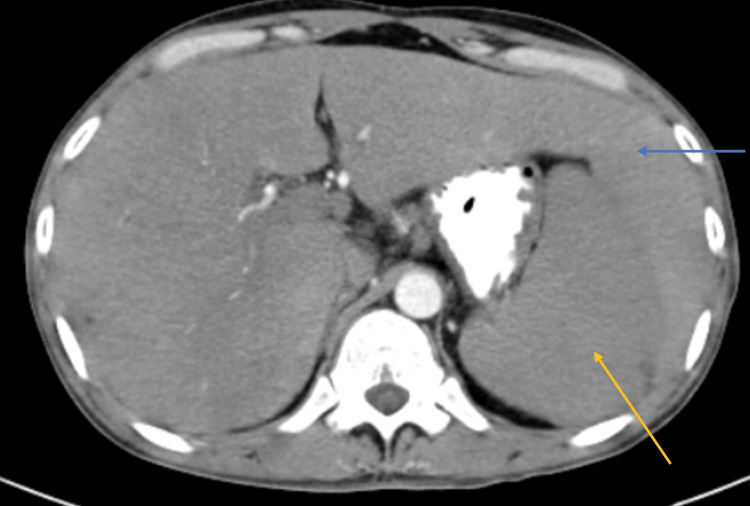
CT of the abdomen showing enlarged left lobe of the liver (blue arrow) enveloping the spleen (yellow arrow) CT: computed tomography

On investigations, the patient was diagnosed with alcoholic hepatitis and not acute pancreatitis. On contrast-enhanced CT scan of the abdomen, there was the presence of an elongated left lobe of the liver, consistent with a beaver tail liver with enlargement of the liver (Figure 1).

Treatment

The patient was started on injectable pantoprazole 40 mg twice a day, injectable ondansetron 4 mg thrice a day, injectable thiamine 100 mg thrice a day, injectable vitamin K 10 mg once a day for three days, injectable acetylcysteine 600 mg thrice a day, and tablet ursodeoxycholic acid 300 mg twice a day. After the initial diagnosis of alcoholic hepatitis and treatment, the patient with a beaver tail variant of the liver was feeling better with declining AST and alanine transaminase (ALT) and was discharged. The patient was asked to follow up after 1 month, and his liver function was normal (ALT: 34 U/L, AST: 48 U/L, alkaline phosphatase: 117 U/L, total bilirubin: 2 mg/dL). The follow-up included regular clinical evaluations and laboratory tests. The patient was advised to maintain a low-fat diet and avoid alcohol consumption to reduce the risk of pancreatitis due to alcohol consumption. Nutritional counseling was provided to help the patient adhere to these dietary recommendations. They were instructed to seek immediate medical attention if any symptoms of complications arose.

## Discussion

Liver anatomic variations are extremely uncommon. The size and shape of the left liver lobe can also vary. However, these variations may appear as accessory or ectopic lobes, more commonly seen in the right lobe [6]. Torsion of the pedunculus connecting to the accessory lobe may manifest as symptoms or an unintentional discovery. There have also been reports of hepatocellular malignancies in these accessory lobes [6]. An anatomical variety known as "beaver tail liver" is a highly unusual inadvertent discovery. This variant can also appear in fibrous connective tissue, the extent of which is from the left lateral edge of the liver to the diaphragm and occasionally contains liver parenchyma. It is distinguished by an extended liver lobe that touches and frequently encircles the spleen as it travels down the front abdominal wall across the midline. Some anatomists know this as appendix fibrosis hepatitis, an anatomic variant named for the fibrous band that connects the liver's left lobe to the diaphragm. The term comes from the fact that it resembles a beaver's tail [7].

 It is composed of normal liver parenchyma. Hence, there is no additional risk of pathology. The beaver tail liver is an uncommon anatomical variation that might be unintentionally found during imaging examinations. While generally asymptomatic, it is essential to recognize this variant to avoid potential complications during surgeries involving the liver or adjacent structures. The elongated left lobe can be mistaken for a pathological mass or an abnormality, leading to unnecessary interventions. It is necessary to distinguish beaver tail liver from splenic mass and hematoma. This condition is challenging to spot on ultrasonography because of its comparable echogenicity to the spleen. On the other hand, the color Doppler research aids in distinguishing between the normal portal and hepatic veins [6,7]. The beaver tail liver is often confused with the "kissing sign" of the liver and spleen that appears on imaging studies when these organs come into contact with one another in individuals with hepatomegaly and splenomegaly. It is essential to identify the "hiding beaver tail liver" variety, in which the lateral part of the left liver lobe angles sharply with the remaining liver and is medially positioned about the visceral surface of the spleen. The literature currently accessible on beaver tail liver is restricted to a few reported cases that describe it as an accidental discovery because of the rarity of this variant. It is interesting to note that because of their larger remaining liver capacity, living liver donors with the "beaver tail" characteristic may recover more safely and have better results [8]. Given the incidental nature of the finding and the lack of symptoms attributable to the beaver tail liver, conservative management was chosen. The patient was educated about the condition and reassured. Regular follow-up was recommended to monitor for any potential complications, although none are expected given the benign nature of the variant [9,10].

We describe a patient in this case study who was first diagnosed with acute pancreatitis based on clinical symptoms such as pain in the upper abdomen, mainly in the epigastrium, and radiating to the back with persistent vomiting. On laboratory investigations, it was revealed that the actual cause was alcoholic hepatitis. Hepatosplenomegaly and a beaver tail appearance of the liver were observed on the CT scan, indicating an uncommon and rare hepatic morphology [11].

## Conclusions

Beaver tail liver, while rare, is an essential anatomical variant that clinicians should be aware of, particularly in the context of hepatic or abdominal surgeries. Incidental findings of this nature should be carefully evaluated to avoid unnecessary surgical interventions. In this case report, we detail the presentation of a patient with known chronic alcohol use who exhibited signs and symptoms initially suggestive of acute pancreatitis. However, further investigation revealed the underlying condition to be alcoholic hepatitis in the context of a beaver tail liver. This rare hepatic anomaly complicated the diagnostic process and exemplified how anatomical variations can lead to potential misdiagnosis and inappropriate management strategies.
